# Altaids: accretionary tectonics writ large

**DOI:** 10.1093/nsr/nwac153

**Published:** 2022-08-02

**Authors:** Jonathan C Aitchison

**Affiliations:** School of Earth and Environmental Sciences, The University of Queensland, Australia

This perspective is written through the lens of someone who, except for a limited but enlightening foray into the region 25 years ago, has largely watched the evolution of thinking and understanding of the Altaids from afar. The area is a hotbed for new ideas and thinking. Many strongly held viewpoints associated with various schools of thought have been applied. Over time, international collaboration has moved thinking towards a consensus view, which hopefully will not stifle valuable originality.

To some, the Altaids represent a unique type of orogenic system, knowledge of which can be applied elsewhere. Others apply models developed elsewhere to interpret evolution of the region over different geological timeframes.

It is approaching 200 years since the German naturalist and explorer Alexander von Humboldt introduced the mountains of Asia to the broader scientific community [1]. This magnificent region of complex and commonly well-exposed geology is a classic accretionary orogen and the type location of Turkic-style orogenesis [2]. Since Seuss [3] introduced the name, this area has been widely referred to as the ‘Altaids’. More recently, the synonymous term ‘Central Asian Orogenic Belt’ has also been used but is perhaps less evocative. This enormous tectonic collage is bounded to the west by the Uralides along the flank of the East European (Russian, Baltic) Craton. In the northeast, the Baikalides mark the boundary with the Siberian (Angora) Craton and on its southern flanks lie the Tarim and North China cratons. The Altaids have significant mineral endowment and are increasingly being explored for potential new economy mineral resources.

Modern plate tectonic concepts arrived late. Applying the 1980s paradigm, Coleman recognized continental, ocean crust, island arc and composite terranes amongst which ophiolitic assemblages featured strongly [4]. In light of more recent ocean drilling discoveries, many of these would now be regarded as having island arc or supra-subduction zone (SSZ) affinities.

In a seminal work [5] and subsequent publications, Şengör and colleagues summarized the tectonostratigraphic architecture of this vast region. They proposed a single convergent margin system associated with the ‘Kipchak Arc’ against which massive accretionary complexes were built from Late Proterozoic to Early Mesozoic time. They posited that the system was oroclinally distorted and later dismembered by strike-slip faulting. This testable hypothesis set the scene for the plenitude of investigations that followed as access to remote areas improved.

At the turn of this century, localized detailed geological investigations saw applications of tectonostratigraphic concepts, and recognition, for the first time [6], of the basalt-chert-tuff succession typical of ocean plate stratigraphy (OPS) in the Kekesayi terrane of West Junggar.

The new century also saw methodologies and instrumentation supporting widespread and systematic acquisition of hitherto scant geochronological data. In particular, development of geochronological techniques led firstly to provision of Nd-Sm age constraints on many of the region's extensive granitoid suites [7]. This was complemented by the introduction of laser ICP-MS instruments that allowed ready determination of U-Pb magmatic ages for zircons. Subsequently, a deluge of data, particularly for basic igneous lithologies and detrital siliciclastic sedimentary rocks, has dominated scientific output. Much of the area has now been investigated in detail and numerous excellent review articles have appeared [8–11]. The evolving literature reflects themes and trends and is variously replete with reports of boninites, large igneous provinces, adakites, oroclines, and more recently, inferences of numerous slab break-off events.

Plate tectonics remains the paradigmatic lens through which data are interpreted, but perhaps regrettably using an overly simplistic approach. Recently, Sengor and colleagues [12] coined a new collective noun and unceremoniously but justifiably dismissed the potential existence of a ‘herd’ of subduction zones. Invocation of a preponderance of new subduction zones to explain every fragment of an arc, ophiolite or accretionary complex belies the true evolutionary complexity of the Altaids. Perhaps in the rush to complete manuscripts, there are times when what is obvious from present day tectonics and plate boundary interactions tends to be overlooked.

Given that students of geology learn that the relative motions of plates are related to non-stationary Euler poles it is curious why static two-dimensional diagrams remain so pervasive amongst tectonic reconstructions. Almost all such cartoons assume trench-normal convergence and overlook the likelihood of lateral translation of large blocks along plate margins. However clear the significance of transform plate boundaries is on satellite imagery, many reconstructions for the Altaids rarely consider palinspastic restoration of paleogeography along such boundaries.

Now that abundant geochronological data, both for primary igneous and for detrital assemblages, are available, the next generation of geologists need to use approaches that will further the understanding and unravelling of the Altaids. Regrettably, the pandemic of detrital zircon analyses and geochemical data lacks context in many areas and largely results from spot-sampling campaigns. The wealth of structural and stratigraphic observations comprising the data otherwise needed to unravel orogenic evolution lags far behind.

A methodology proven in other orogenic systems such as the European Alps, which considers both potential strike-slip displacement and long-distance low angle over-thrusting of tectonic assemblages, might prove extremely useful. Curiously, although the existence of regional-scale fold structures such as the Kazakhstan and Tuva-Mongol oroclines is widely postulated [5,12,13], speculation about the three-dimensional architecture of the Altaids remains limited. Deep passive seismic profiling to Moho depths has recently become available and sheds light on crustal scale architecture [14]. However, given their utility in testing the veracity of geological mapping and demonstrated effectiveness in unravelling the evolution of other complex tectonic systems, lack of recourse to large-scale cross sections is surprising. Few, if any, ‘Alpine-style’ cross sections redolent with deep structural detail that shed light on the architectural anatomy of the Altaids exist.

Relatively little consideration has been given to the opening of early oceanic spaces and the location of former continent–ocean boundaries. Whether or not some rock assemblages interpreted as ophiolites might be relics of former ocean–continent transitions (OCTs) and/or oceanic metamorphic complexes (OCCs) is worth further investigation. Accompanied by structural data, detailed consideration of OPS successions and age differences between initial ocean ridge sedimentation and later trench-fill sedimentation as oceanic crust enters a subduction zone, offers the potential to unravel histories of now-subducted oceanic plates in oceans that closed, leading to the Altaids orogenesis. As magmatic fronts should have been located above where subducting slabs attained magmagenic depths, variations in their locations potentially record changes in slab dip, locations of slab windows and other intricacies of plate subduction. Much remains to be tested, resolved and retested. The future for investigations of the Altaids is arguably more exciting than ever.
